# Fibrodysplasia Ossificans Progressiva and Pregnancy: A Case Series and Review of the Literature

**DOI:** 10.1155/2022/9857766

**Published:** 2022-09-16

**Authors:** Alexandra D. Forrest, Danielle M. Vuncannon, Jane E. Ellis, Zvi Grunwald, Frederick S. Kaplan

**Affiliations:** ^1^Department of Gynecology and Obstetrics, Emory University School of Medicine, 80 Jesse Hill Jr. Drive SE, Atlanta, GA 30303, USA; ^2^Department of Anesthesiology, Sidney Kimmel Medical College at Thomas Jefferson University Hospital, Suite 515, 1020 Walnut Street, Philadelphia, PA 19107, USA; ^3^Department of Orthopedic Surgery and Medicine, The Perelman School of Medicine, University of Pennsylvania, Penn Musculoskeletal Center, Suite 600, 3737 Market Street, Philadelphia, PA 19104, USA

## Abstract

**Objective:**

To evaluate maternal and fetal outcomes in pregnant patients with fibrodysplasia ossificans progressiva (FOP; OMIM#135100), an ultrarare genetic disorder characterized by progressive heterotopic ossification of soft tissues and cumulative disability.

**Methods:**

This is a retrospective case series of three patients with FOP who were admitted to Grady Memorial Hospital in Atlanta, Georgia, from to February 2011 to July 2021.

**Results:**

Three women delivered preterm infants at our institution. These cases posed unique anesthetic and obstetric technical challenges, particularly when securing the airway and performing cesarean delivery. Importantly, each patient received perioperative glucocorticoids for prevention of further heterotopic ossification.

**Conclusion:**

FOP is a unique clinical diagnosis encountered by obstetricians and requires multidisciplinary management for optimal outcomes.

## 1. Introduction

Fibrodysplasia ossificans progressiva (FOP; OMIM# 135100) is an ultrarare genetic disorder characterized by progressive heterotopic ossification (HO) of soft tissues including muscles, tendons, ligaments, fascia, and aponeuroses leading to cumulative disability. HO usually begins in childhood, resulting in progressive limitation of thoracic volume and immobilization of the chest wall, limbs, and jaw by early adulthood [1]. Median life expectancy for patients with FOP is 56 years. The international prevalence of disease is approximately 1 in 1 million births [2]. The genetic inheritance pattern is autosomal dominant; however, most cases are the result of a spontaneous mutation in the activin A receptor type I/activin receptor-like kinase 2 (*ACVR1/ALK2*) gene [3]. Mutation of this gene results in dysregulation of the bone morphogenetic protein signaling pathway. Specifically, ALK R206H enhances chondrogenesis and contributes to episodes of HO [4]. Heterotopic bone formation often occurs secondary to soft tissue injury or may occur spontaneously. HO is episodic; disability is cumulative [5].

Diagnosis is clinical and is based on skeletal malformations including malformed great toes, soft tissue swelling, and progressive HO [6, 7]. Genetic diagnosis is considered confirmatory and is based on identification of the *ACVR1/ALK2* gene mutation [7]. Misdiagnosis is unfortunately quite common and occurs in over 80% of patients [8]. Management is focused on early diagnosis, avoidance of injury or iatrogenic harm, symptomatic treatment of flares with glucocorticoids, and optimization of residual function [9].

Pregnancy is extremely rare among persons with FOP and poses unique maternal and fetal risks [10–13]. Previously reported risks include those of HO with rapid progression of disease, worsening pulmonary status due to progressive thoracic volume reduction and limited chest wall compliance, spontaneous abortion, preterm delivery, cesarean birth, venous thromboembolism, wound formation, and 50% risk of fetal inheritance. A literature search performed on PubMed in December 2021 using the keywords fibrodysplasia ossificans progressiva and pregnancy yielded 15 results. All articles were reviewed for nonredundancy and the presence of specific cases of FOP and pregnancy. To our knowledge, only five instances of childbirth among patients with FOP have been described in the literature. In this case series, we describe the management and outcomes of three additional patients with FOP who delivered at our institution. Written consent for publication was obtained from all patients or their next of kin.

## 2. Cases

### 2.1. Patient 1

A 25 yo G2P0010 with classic FOP (inherited from her father) and severe restrictive pulmonary disease presented to our institution at 22 weeks gestation for initial consultation. Her obstetric history was notable for a missed abortion at 8 weeks gestation which was managed with misoprostol. The patient was evaluated by the perinatology, anesthesiology, and neonatology services and was counseled about the risks of her pregnancy.

She was transferred from an outside hospital to our care at 25 weeks gestation because of preterm labor. She received oral dexamethasone for fetal lung maturation and indomethacin for tocolysis and her contractions subsided. On hospital day five, she developed recurrent uterine contractions. Her cervical exam progressed to six cm dilation, and the decision was made to proceed with urgent delivery. She was transferred to the operating room (OR) where she underwent a successful awake nasotracheal fiberoptic intubation by anesthesiology with otolaryngology in the OR in case of the need for emergent tracheostomy. The patient underwent a classical cesarean section followed by bilateral tubal ligation. She delivered a viable male infant weighing 750 grams with Apgar scores of 1 and 8 at 1 and 5 minutes, respectively. The delivery was complicated by a postpartum hemorrhage of 2800 mL secondary to uterine atony and inadequate hemostasis at the hysterotomy because of the vertical orientation and need for multilayer closure. She was transfused with three units of packed red blood cells (pRBCs) and two units of fresh frozen plasma intraoperatively.

Postoperatively, she was transferred to the intensive care unit (ICU) under mechanical ventilation and sedation. On postoperative day one, the trachea was extubated uneventfully in the OR under the supervision of anesthesiologists and otolaryngologists. During her postpartum course, she required one additional unit of pRBC for the treatment of anemia and diuresis for pulmonary edema due to volume overload. She was discharged home on postoperative day six, seen for follow-up at three and seven weeks postpartum, and was doing well. Her ability to care for her infant, however, was significantly limited by her FOP. Physical examination and genetic testing confirmed that her infant was not affected by FOP.

### 2.2. Patient 2

A 27 yo sister of patient 1, G1P0 with classic FOP, restrictive pulmonary disease, and anxiety, presented to our institution at 18 weeks gestation. As with her sister, patient 2 inherited FOP from her father. She was admitted to the antepartum service for consultation with perinatology, endocrinology, anesthesiology, otolaryngology, neonatology, pulmonology, cardiology, and nutrition.

Her antepartum course was complicated by several presentations for threatened preterm labor, gestational hypertension, and fetal growth restriction (FGR). She received two courses of oral dexamethasone for fetal lung maturation. At 30 weeks gestation, she presented with uterine contractions. Preterm labor was ruled out; however, she was readmitted for coordination of care. On hospital day 32, at 34 weeks gestation, she was diagnosed with preterm premature rupture of membranes and preterm labor and was prepared for urgent cesarean section. She underwent nasotracheal fiberoptic intubation followed by a classical cesarean section and bilateral tubal ligation and delivered a viable male infant. The child weighed 1610 g with Apgars 1, 1, and 6 at 1 minute, 5 minutes, and 10 minutes of life. Numerous calcifications were noted intraoperatively in the myometrium, omentum, and rectus musculature. The myometrial calcifications made repair of the hysterotomy challenging and required myometrial wedge resection to achieve closure. The delivery was complicated by a postpartum hemorrhage of 2000 mL secondary to inadequate hemostasis at the hysterotomy due to the above anatomic challenges. She was transfused with four units pRBCs intraoperatively.

**Figure 1:**
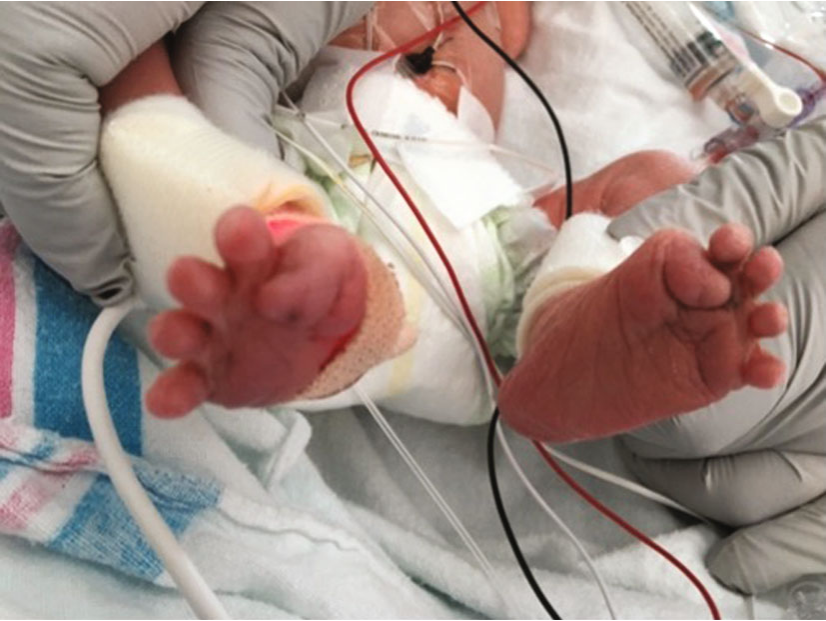
This figure depicts the malformed great toes of patient 2's baby. Based on physical exam, there was high clinical suspicion for FOP, and subsequent testing for the ACVR1/ALK2 gene mutation confirmed a diagnosis of classic FOP.

Postoperatively, she received two additional units of pRBC for anemia. She was diagnosed with preeclampsia with severe features on postoperative day one based on elevated blood pressures and visual disturbances, specifically scotomata and blurred vision (Table 1). She received magnesium sulfate for 24 hours without issue. She was discharged in stable condition on postoperative day nine. She was seen for follow-up at four weeks postpartum at which time she had multiple new-onset pressure ulcers along her posterior thighs and was referred to the wound care team. Her infant was diagnosed with classic FOP based on malformed great toes and confirmatory genetic testing (Figure 1).

### 2.3. Patient 3

**Figure 2:**
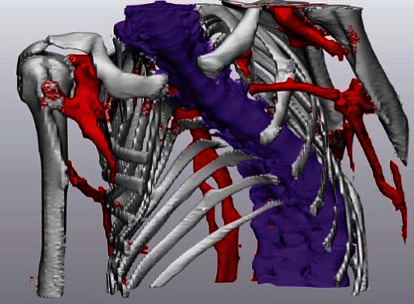
This image is a 3D model of patient 3's computed tomographic chest scan performed at the time of her initial presentation to our care. The sternum is absent to allow for visualization of the path of the spine, which is depicted in purple. Heterotopic bone formation is portrayed in red.

An 18 yo G2P0010 with classic FOP, severe restrictive pulmonary disease, chronic hypertension, and seizure disorder, presented to our care at 15 weeks gestation (Figure 2). Her obstetric history was notable for a miscarriage. The etiology of her FOP was a spontaneous genetic mutation as neither of her parents tested positive for the FOP genetic mutation. She was admitted to our antepartum service for consultation with perinatology, endocrinology, anesthesiology, otolaryngology, pulmonology, neonatology, and physical therapy.

Her antepartum course was complicated by FGR, FOP flare-ups in the hips and lumbosacral area, and worsening pulmonary status. She was readmitted to the antepartum service at 33 weeks gestation for close monitoring and planned preterm cesarean section. At the time of admission, she received oral dexamethasone for fetal lung maturation. At 34 weeks gestation, she underwent a scheduled cesarean delivery following a nasotracheal fiberoptic intubation. The intubation was challenging due to distorted airway anatomy and copious secretions and required three attempts. She delivered a male infant weighing 1810 g with Apgar scores of 1 and 8 at 1 and 5 minutes. There was difficulty with delivery due to an adhesive band in the lower uterine segment; therefore, the initial low transverse uterine incision was extended to a T-incision, and breech extraction was performed. The delivery was also complicated by postpartum hemorrhage of 1031 mL secondary to inadequate hemostasis at the hysterotomy due to the above anatomic distortions.

**Figure 3:**
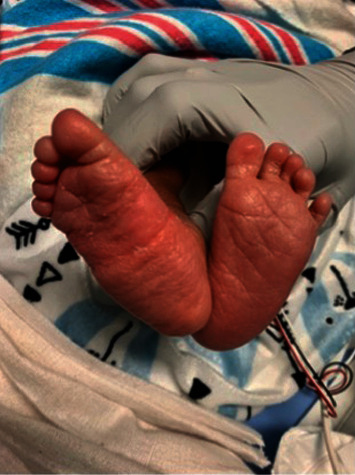
This figure depicts the feet of the baby born to patient 3 in this case series. The great toes appear normal. Genetic testing was performed immediately following delivery and revealed absence of the ACVR1/ALK2 gene mutation.

The patient initially experienced an uncomplicated postoperative course. On postoperative day three, however, she developed new-onset severe hypertension and was diagnosed with preeclampsia with severe features (Table 1). She received a loading dose of magnesium sulfate, but a maintenance dose was not administered due to concerns of precipitating an FOP flare-up with frequent venipunctures. On hospital day four, she developed tachycardia and tachypnea and refused enoxaparin. A heparin drip was initiated but a computed tomographic pulmonary embolus study was negative, and the heparin was discontinued. The remainder of her postpartum course was uncomplicated. She was discharged home with progesterone-only pills for contraception. She was seen for follow-up at three and five weeks postpartum and was doing well. Her infant had an uncomplicated course; his great toes were normal, and genetic testing ruled-out a diagnosis of FOP (Figure 3).

## 3. Discussion

Prior to conception, it is imperative that persons with FOP be educated about the risks associated with pregnancy. They should be informed of the risks of heterotopic ossification with rapid progression of disease, worsening pulmonary status due to limited thoracic volume and chest wall compliance, spontaneous abortion, preterm delivery, cesarean birth, venous thromboembolism, wound formation, 50% risk of fetal inheritance, and maternal and fetal death [10]. For those individuals who are sexually active but do not desire pregnancy, appropriate contraception should be prescribed. Due to risk of heterotopic ossification associated with intramuscular injections, Depo-Provera should be avoided [14].

At the time of conception, patients with FOP should be counseled about risks of pregnancy, and termination should be offered. Should a patient desire continued pregnancy, it is essential that they be referred to a tertiary care center where multidisciplinary care can be coordinated with obstetrics, perinatology, anesthesiology, pulmonology, otolaryngology, endocrinology, neonatology, nursing, and physical therapy. Importantly, care should also be comanaged by an FOP expert [15].

Etiologies for preterm birth in this population include spontaneous preterm labor, iatrogenic preterm birth due to worsening maternal status, and fetal distress [10]. It is imperative that corticosteroids be administered in the event of anticipated preterm delivery. Due to the risk of FOP flare-up associated with intramuscular injections, dexamethasone should be administered orally instead of by intramuscular injection [16].

For some but not all patients, pregnancy exacerbates symptoms of FOP. This can result in disease progression and further limitation of mobility due to new-onset ossification. For some, it results in FOP flare-ups which should promptly be treated with corticosteroids. For others, restrictive pulmonary dysfunction may become more pronounced due to pulmonary compression by the increasing intra-abdominal volume and reduced chest wall compliance. It is imperative that an anesthesiologist, pulmonologist, and FOP expert be available for consultation in these scenarios.

In the event of delivery, vaginal delivery is contraindicated due pelvic deformity, hip contractures or ankylosis, and spinal fusion [15]. Cesarean delivery is therefore the modality of choice, although there is the potential for heterotopic ossification to occur at the incision site. Due to risk of disease flare-up associated with surgery, prophylactic prednisone at a dose of 1-2 mg/kg daily should be administered at the time of delivery and for three days postpartum [15]. Corticosteroids are a well-established therapy for flare-ups, and therefore, their use has been extended to prophylaxis, particularly in the setting of surgery when there is high risk for HO at the operation site [17]. Patients should also be carefully positioned with the assistance of additional padding to prevent pressure sores and neuropathy. Intraoperatively, the delivery team should be prepared for a challenging procedure due to distorted anatomy. Postoperatively, patients should receive anticoagulation with low molecular weight heparin due to risk of venous thromboembolism [15].

Patients with FOP are not candidates for regional anesthesia due to fusion of the lumbar spine, hips, and sacroiliac joints. Needle insertion when attempting epidural or spinal access may also precipitate heterotopic bone formation and should be avoided. Cervical spine fusion, ankylosis of the temporomandibular joints, thoracic insufficiency syndrome, restrictive chest wall disease, and sensitivity to oral trauma complicate airway management and anesthesia and pose life-threatening risks. Anesthesia with an awake intubation by nasotracheal fiberoptic technique should be performed [18, 19]. Importantly, a surgeon should be available to perform an emergency tracheostomy in case of a failed intubation.

Interestingly, postpartum hemorrhage, preeclampsia with severe features, and FGR occurred in at least two of our three cases. While these have not been previously described in pregnancies that occurred at other institutions, consideration should be given to the potential increased risks of these events in this patient population. The increased risk for postpartum hemorrhage may be secondary to general anesthesia, distorted anatomy, and use of vertical uterine incision in some cases [20]. Uterotonics may be administered, though care should be taken to avoid intramuscular injections and instead consideration should be given to intrauterine or intravenous administration. There is no known link between FOP and chronic hypertension; however, ACVR2A, a cell surface receptor associated with ACVR1, has recently been implicated as a potential genetic precursor to preeclampsia [21, 22]. Whether there may be a genetic component of FOP that predisposes these patients to develop hypertensive disorders of pregnancy remains to be determined. A potential link with FGR could be due to space limitations for fetal growth secondary to abdominal wall ossification or chronic hypoxia associated with restrictive lung disease.

Pregnancy with FOP is a rare clinical scenario, and therefore, data on associated risks and optimal management is limited. Ultimately, should a patient choose to continue pregnancy, they should be referred to a tertiary care center where a multidisciplinary team can be formed to allow for coordination of care and management of optimal outcomes.

## Figures and Tables

**Table 1:** Clinical criteria of patients diagnosed with hypertensive disorders of pregnancy.

Patient	Diagnosis	Gestational age at diagnosis	Diagnostic criteria	Therapeutic agents
Patient 2	Preeclampsia with severe features	Postpartum day 1	Severe range blood pressures, visual disturbances	Magnesium, nifedipine 30 mg XL daily
Patient 3	Chronic hypertension with superimposed preeclampsia with severe features	Postpartum day 3	Severe range blood pressures	Magnesium, labetalol 600 mg twice daily

## Data Availability

The data used to support the findings of this study are included within the article.
